# Irisin Contributes to the Hepatoprotection of Dexmedetomidine during Intestinal Ischemia/Reperfusion

**DOI:** 10.1155/2019/7857082

**Published:** 2019-05-02

**Authors:** Xin Fan, Juan Du, Mao-Hua Wang, Jia-Man Li, Bo Yang, Ye Chen, Jun-Chao Dai, Chunxiang Zhang, Jun Zhou

**Affiliations:** ^1^Department of Anesthesiology, Affiliated Hospital of Southwest Medical University, Luzhou, China; ^2^Department of Anesthesiology, The First People's Hospital of Ziyang, Ziyang, China; ^3^Department of Traditional Chinese Medicine, Affiliated Hospital of Southwest Medical University, Luzhou, China; ^4^Department of Biomedical Engineering, School of Medicine, University of Alabama at Birmingham, Birmingham, Alabama, USA

## Abstract

Intestinal ischemia/reperfusion (I/R), which is associated with high morbidity and mortality, is also accompanied with abnormal energy metabolism and liver injury. Irisin, a novel exercise-induced hormone, can regulate adipose browning and thermogenesis. The following study investigated the potential role of dexmedetomidine in liver injury during intestinal I/R in rats. Adult male Sprague–Dawley rats underwent occlusion of the superior mesenteric artery for 90 min followed by 2 h of reperfusion. Dexmedetomidine or irisin-neutralizing antibody was intravenously administered for 1 h before surgery. The results demonstrated that severe intestine and liver injuries occurred during intestinal I/R as evidenced by pathological scores and an apparent increase in serum diamine oxidase (DAO), aspartate aminotransferase (AST), alanine aminotransferase (ALT), and lactate dehydrogenase (LDH) levels. In addition, the hepatic irisin, cleaved caspase-3, Bax, and NLRP3 inflammasome components (including NLRP3, ASC, and caspase-1), protein expressions, apoptotic index, reactive oxygen species (ROS), malondialdehyde (MDA), myeloperoxidase (MPO), tumor necrosis factor- (TNF-) *α*, and interleukin- (IL-) 6 levels increased; however, the serum irisin level and hepatic Bcl-2 protein expression and superoxide dismutase (SOD) activity decreased after intestinal I/R. Interestingly, dexmedetomidine could reduce the above listed changes and increase the irisin levels in plasma and the liver in I/R rats. Dexmedetomidine-mediated protective effects on liver injury and NLRP3 inflammasome activation during intestinal I/R were partially abrogated via irisin-neutralizing antibody treatment. The results suggest that irisin might contribute to the hepatoprotection of dexmedetomidine during intestinal ischemia/reperfusion.

## 1. Introduction

Intestinal ischemia–reperfusion (I/R) is a common pathophysiological phenomenon commonly present in various kinds of life-threatening conditions, such as cardiopulmonary bypass, vascular embolism, and small bowel transplantation [1–5]. Moreover, intestinal I/R is vital in the progressively deteriorative state of an illness. Intestinal I/R results in an abnormal energy metabolism, intestinal local tissue necrosis, and apoptosis. Moreover, it triggers a systemic inflammatory response, accompanied by damage to remote organs including the brain, liver, lung, and kidney, and it can eventually lead to death [6–10].

The liver is one of the most vulnerable organs after intestinal I/R since the liver and the intestine share the common anatomical pathway, such as coupled vasculature [9, 11]. Liver tissue contains many biochemical elements that are involved in free radical scavenging and inflammatory reactions. Currently, the activation, adhesion, and release of toxic substances from polymorphonuclear neutrophils (PMNs) are believed to be key factors in the development of the damage induced by intestinal I/R. The excessive generation of reactive oxygen species (ROS), cytokine/chemokine generation, activated complement, and neutrophil activation is all involved in injury to remote organs following intestinal I/R [1, 6]. Those cause liver cell edema, bacterial and inflammatory cell infiltration, and significant increases in the levels of oxygen-free radicals and lipid peroxidation products in the liver tissue [9, 11]. However, the mechanism of intestinal I/R-induced liver injury has not yet been fully elucidated.

Irisin, a novel skeletal muscle cell-derived myokine, was initially discovered as a cleavage product of the extracellular portion of type I membrane protein fibronectin type III domain containing 5 (FNDC5). It was induced by physical exercise to increase the energy expenditure [12]. Interestingly, irisin has been found to be almost ubiquitously present in almost all biological tissues, such as the skeletal muscle, liver, and brain tissues since the discovery of muscle-secreted hormone. Irisin could reduce inflammation, oxidative stress, and apoptosis in many different models [13, 14]. Therefore, recent studies have demonstrated that irisin might be used as a treatment for metabolic disorders and cardiovascular diseases by inducing the white adipose tissue browning [14, 15]. Irisin is considered to have a pivotal role in protecting the heart and brain against ischemia and reperfusion injury [15, 16]. Additionally, irisin could also be used for atherosclerotic vascular diseases in diabetes due to ameliorating atherosclerosis [17]. Nevertheless, to date, the roles of irisin in intestinal diseases are far from being completely understood. The nucleotide binding and oligomerization domain-like receptor family pyrin domain-containing 3 (NLRP3) inflammasome, as an important component of innate immunity, plays a pivotal role in the process of immune response and inflammatory diseases. It can be activated by multiple types of pathogens or danger signals. NLRP3 inflammasome mediates the developments of many diseases, such as type 2 diabetes, ischemia reperfusion injury, sepsis, and atherosclerosis [18–20]. Therefore, as the core of inflammatory response, NLRP3 inflammasome may provide new targets for the treatment of various inflammatory diseases.

Dexmedetomidine is a new drug that has been commonly used in clinical studies over recent years. It is also preferred by various clinical departments including anesthesia departments and intensive care units because of its analgesic, sedative, and antidepressant effects [21, 22]. Previous studies have indicated that dexmedetomidine also has protective effects against the injuries of organs in animal models [23–26]. Nevertheless, studies on the mechanisms underlying its protective effects are inadequate. The effects of dexmedetomidine on intestinal I/R-mediated liver injury and the potential role of irisin in the hepatoprotective effects of dexmedetomidine remain unclear.

Based on previous findings, we hypothesized that dexmedetomidine could attenuate intestinal I/R-mediated liver lesions by inhibiting NLRP3 inflammasome activation and irisin might play a crucial role in the beneficial effects of dexmedetomidine on the liver injury, inflammation, and oxidative stress. Therefore, the present study was designed to confirm the aforementioned hypothesis and to elucidate the protective mechanisms in a rat model.

## 2. Material and Methods

### 2.1. Animals and Treatment

Adult male Sprague–Dawley rats, weighing 230-260 g, were provided by the Laboratory Animal Center of Southwest Medical University. All rats were housed in animal quarters under controlled temperature and humidity with a light/dark cycle of 12/12 hr and received food and water ad libitum. Food was removed 12 h before the surgery, but the rats had free access to water. In addition, all animal studies were done in compliance with the approval of the institutional animal care and use committee of Southwest Medical University, and all experiments were conducted according to the National Institutes of Health guidelines.

In experiment 1, the rats were randomly divided into four groups (*n* = 8 per group): sham-operated (sham group), intestinal I/R (I/R group), 2.5 *μ*g/(kg·h) dexmedetomidine (I/R+Dex1 group), and 5 *μ*g/(kg·h) dexmedetomidine (I/R+Dex2 group). Intestinal I/R was established according to our previous study [6]. Briefly, rats were anesthetized with ketamine/xylazine (10 : 1, 100 mg/mL, 0.1 mL per 100 gm of the rat's body weight was injected intraperitoneally). The superior mesenteric artery (SMA) was occluded for 90 min using a noninvasive artery clip; then the clip was released for 2 h reperfusion. However, the SMA was isolated only in the sham-operated group, but not clamped. The rats in the I/R+Dex1 and I/R+Dex2 groups were continuously infused with dexmedetomidine (Orion Pharma, Turku, Finland) (2.5 and 5 *μ*g/(kg·h)) via the tail vein for 1 h before the intestinal I/R experiment. The rats in the sham and I/R group were continuously administered with the same volume of vehicle (0.9% normal saline). A total of eight rats from each group were randomly selected to obtain blood, intestine, and liver tissue samples for further analysis after 2 h reperfusion.

In experiment 2, the rats were randomly divided into seven groups (*n* = 8 per group): sham group, I/R group, I/R+control IgG group, I/R+irisin-neutralizing antibody (NA) group, I/R+Dex group, I/R+Dex+control IgG group, and I/R+Dex+irisin NA group. Dexmedetomidine (5 *μ*g/(kg·h)) was administered for 1 h as previously described (see experiment 1); the rats in the I/R+Dex group were administered with dexmedetomidine; the rats in the I/R+control IgG and I/R+Dex+control IgG groups were infused with dexmedetomidine and nonimmune control IgG; and I/R+irisin NA and I/R+Dex+irisin NA rats received a combination of dexmedetomidine and irisin-neutralizing antibody (30 *μ*g per rat, Phoenix Pharmaceuticals) [16]. The control IgG or irisin-neutralizing antibody was continuously administered alone by another tail vein for 1 h before surgery. The rats were evaluated for morphological examination and other biochemical parameters at 2 h after I/R.

### 2.2. The Morphological Assessment of the Liver and Intestine

The liver and intestine tissues embedded in paraffin were cut into 4 *μ*m-thick sections. The sections were stained with hematoxylin and eosin and were observed. Next, the extent of injury of the liver was assessed using Suzuki's classification by two experienced pathologists who were blinded to the study [27]. The degree of intestinal damage was evaluated independently using Chiu's scoring method as previously described [6, 28]. Five randomly chosen fields from each rat were evaluated and averaged.

### 2.3. Measurement of Serum DAO, AST, ALT, and LDH Levels

Blood samples were centrifuged at 3000 rpm for 10 min, and the obtained supernatant was stored at −80°C. Diamine oxidase (DAO), as an enzyme, is synthesized primarily in gastrointestinal mucosal cells. The serum diamine oxidase level has been used as an indicator of the integrity and functional mass of the intestinal mucosa [28]. In the present study, serum DAO was detected using a chemical assay kit (Nanjing Jiancheng Bioengineering Institute, Nanjing, China) with a spectrophotometer according to the manufacturer's protocol. Results were expressed as unit per liter serum. In addition, a medical laboratory technician using the ADVIA 2300 automatic analyzer (Siemens Healthcare, Erlangen, Germany) measured the aspartate aminotransferase (AST), alanine aminotransferase (ALT), and lactate dehydrogenase (LDH) levels in the serum.

### 2.4. Detection of Serum Irisin Levels and Irisin, TNF-*α*, and IL-6 Levels in Liver Tissues

Serum and hepatic irisin levels were measured with a commercial ELISA kit (Phoenix Pharmaceuticals, Burlingame, CA, USA) [29]. Samples were evaluated with an ELX 800 ELISA reader at 450 nm absorbance. The experiments were performed in triplicate. In addition, the results were expressed as ng/mL or pg/mg protein.

Liver tissues were homogenized with ice-cold normal saline. The homogenates were centrifuged at 4000 rpm at 4°C for 10 min, and then the supernatants were transferred into fresh tubes for further analysis. The levels of TNF-*α* and IL-6 in liver tissues were determined using commercially available enzyme-linked immunosorbent assay kits (R&D Systems Inc., MN, USA) according to the manufacturer's protocol.

### 2.5. Detection of Myeloperoxidase Activity in Liver Tissues

Myeloperoxidase is a biochemical quantitative marker of the presence of neutrophils in the liver. The liver sample was weighed and homogenized. The homogenate was freeze-thawed twice and then centrifuged at 13,000*g* for 5 min. The resulting supernatant was used for assessing MPO activity with a rat MPO assay kit (Nanjing Jiancheng Bioengineering Institute, Nanjing, China) according to the manufacturer's protocol [30].

### 2.6. Measurements of ROS, MDA Levels, and SOD Activity in Liver Tissues

The tissue homogenate of the liver was prepared, and the supernatant was collected after centrifugation. Hepatic ROS levels were detected in supernatants using the dye 2′,7′-dichlorofluorescein diacetate as previously described [6]. MDA levels and SOD activities were measured using commercial kits (Nanjing Jiancheng Bioengineering Institute, Nanjing, China) according to the manufacturer's protocol [30].

### 2.7. Western Blotting Analyses

The total protein of hepatic tissue was extracted, and the protein concentration was determined using a bicinchoninic acid assay reagent (Pierce Chemical Company, IL, USA). Each 40 *μ*g aliquot of protein was separated by 8% sodium dodecyl sulfate–polyacrylamide gel electrophoresis. The protein was electroblotted onto polyvinylidene difluoride membranes (Amersham Biosciences, NJ, USA). Membranes were blocked using 5% nonfat milk for 2 h at room temperature. Then, samples were then incubated with primary antibodies for cleaved caspase-3 (1 : 1000), Bax (1 : 400), Bcl-2 (1 : 400), NLRP3 (1 : 300), ASC (1 : 200), caspase-1 (1 : 200) (Santa Cruz Biotechnology, Inc., CA, USA), irisin (1 : 500; Phoenix Pharmaceuticals, CA, USA), and GAPDH (1 : 1000; Cell Signaling Technology, Inc., MA, USA) at 4°C overnight. The membranes were then washed three times with TBST and incubated for 1 h at 37°C with anti-rabbit IgG or anti-mouse IgG secondary antibodies (1 : 2000; ZSGB-Bio, Beijing, China). Finally, immunoreactivity was detected using enhanced chemiluminescence reagent.

### 2.8. Determination of Hepatocyte Apoptosis by the TUNEL Method

The apoptotic cells were assessed using a commercial assay kit (Roche, IN, USA) [6]. The liver tissue sections underwent strict dewaxing. Next, 50 *μ*L of TUNEL reaction mixture was added, and the sections were incubated at 37°C for 60 min in a dark humidified atmosphere. After stained again, apoptotic hepatocytes were observed under the fluorescence microscope at high-magnification (400x) field of vision. Five fields of vision were randomly selected to observe and calculate the apoptosis index and hence evaluate the hepatocyte apoptosis.

### 2.9. Statistical Analysis

Statistical analysis was performed using the SPSS 19.0 (SPSS for Windows, Chicago, IL) software. The data were expressed as mean ± standard deviation. One-way ANOVA with the LSD post hoc test was used to examine differences. A separate two-way ANOVA examined the effects of irisin-neutralizing antibody on the levels of serum and hepatic irisin. Biochemical assays were performed in triplicate for each specific sample. Therefore, all data points are means of numbers that themselves are means of triplicate measurements for these parameters. *P* < 0.05 was considered statistically significant.

## 3. Results

### 3.1. Dexmedetomidine Improved the Intestinal and Hepatic Pathological Injury

**Figure 1:**
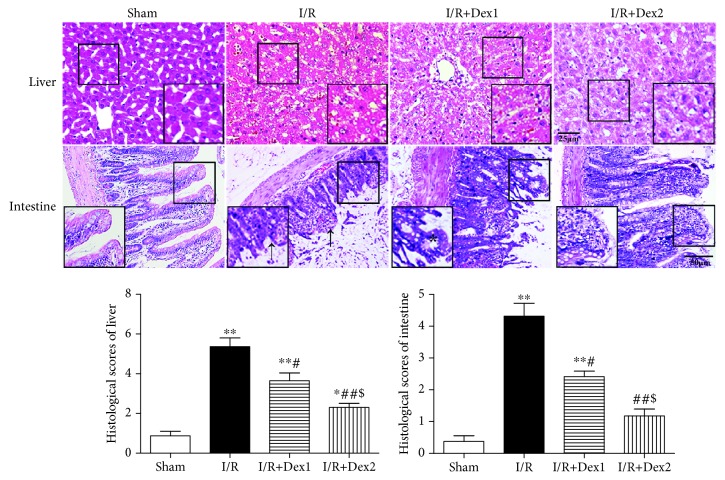
Effect of dexmedetomidine on histologic injuries of the liver and intestine. Dexmedetomidine reduced the histologic injuries of the liver and intestine after intestinal I/R in different groups (hematoxylin and eosin staining, scale bar = 25 or 50 *μ*m; inset, magnified photographs). Black arrows indicate denuded villi and haemorrhage, and black asterisks indicate Gruenhagen's space. The results were presented as the mean ± standard deviation (*n* = 8; ^∗^*P* < 0.05, ^∗∗^*P* < 0.01 vs. sham group; ^#^*P* < 0.05; ^##^*P* < 0.01 vs. I/R group; ^$^*P* < 0.05 vs. I/R+Dex1 group).

These findings suggested that dexmedetomidine could significantly protect rats against intestinal I/R. Notedly, no obvious change in the intestine or liver was observed in the sham group. Yet, severe damage of the liver (A) and intestinal mucosa (B) was observed in the I/R group. The pathological scores in the I/R group were significantly higher than those in the sham group (*P* < 0.01). The scores in the I/R+Dex1 and I/R+Dex2 group were significantly decreased compared with the I/R group (*P* < 0.05). In addition, the scores were lower in the I/R+Dex2 group than those in the I/R+Dex1 group (*P* < 0.05) (Figure 1).

### 3.2. Dexmedetomidine Improved Serum DAO and Liver Dysfunction

**Figure 2:**
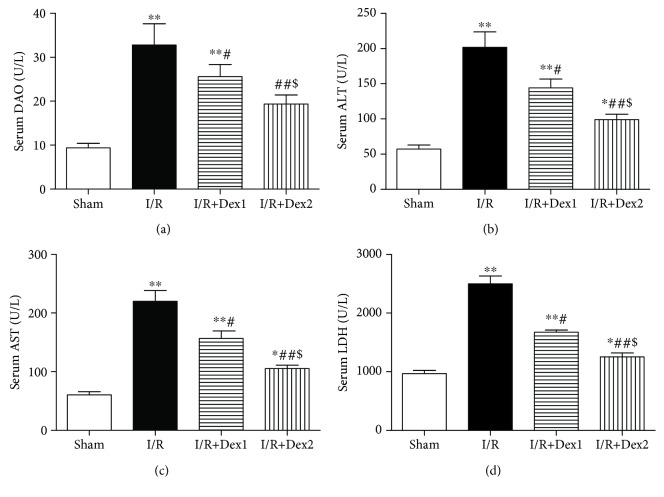
Dexmedetomidine improved serum DAO and liver dysfunction. Dexmedetomidine treatment decreased (a) DAO, (b) ALT, (c) AST, and (d) LDH levels and improved intestinal I/R-induced liver dysfunction. The results were presented as the mean ± standard deviation (*n* = 8; ^∗^*P* < 0.05, ^∗∗^*P* < 0.01 vs. sham group; ^#^*P* < 0.05; ^##^*P* < 0.01 vs. I/R group; ^$^*P* < 0.05 vs. I/R+Dex1 group).

Furthermore, the levels of DAO, ALT, AST, and LDH dramatically increased in the I/R group compared with the sham group (*P* < 0.01). Dexmedetomidine decreased these levels in the dexmedetomidine treatment groups compared with the I/R group (*P* < 0.05). Moreover, the levels of DAO, ALT, AST, and LDH were lower in the I/R+Dex2 group than those in the I/R+Dex1 group (*P* < 0.05) (Figures 2(a)–2(d)).

### 3.3. Dexmedetomidine Decreased TNF-*α*, IL-6, and MPO in Liver Tissues

**Figure 3:**
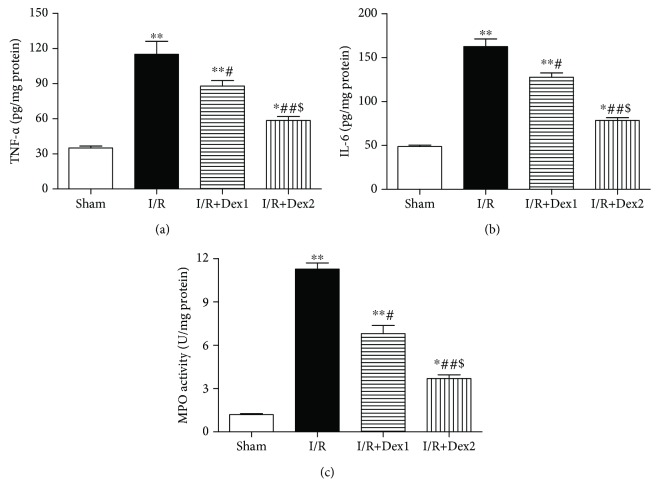
The effect of dexmedetomidine on TNF-*α*, IL-6, and MPO in liver tissues. Dexmedetomidine treatment decreased the (a) TNF-*α*, (b) IL-6, and (c) MPO levels after intestinal I/R in different groups. The results were presented as the mean ± standard deviation (*n* = 8; ^∗^*P* < 0.05, ^∗∗^*P* < 0.01 vs. sham group; ^#^*P* < 0.05; ^##^*P* < 0.01 vs. I/R group; ^$^*P* < 0.05 vs. I/R+Dex1 group).

Additionally, the levels of TNF-*α* (a), IL-6 (b), and MPO (c) in liver tissues obviously increased in the I/R group compared with the sham group (*P* < 0.01). Nevertheless, dexmedetomidine decreased the levels of TNF-*α*, IL-6, and MPO (*P* < 0.05) (Figure 3).

### 3.4. Dexmedetomidine Reduced Hepatic Oxidative Stress

**Figure 4:**
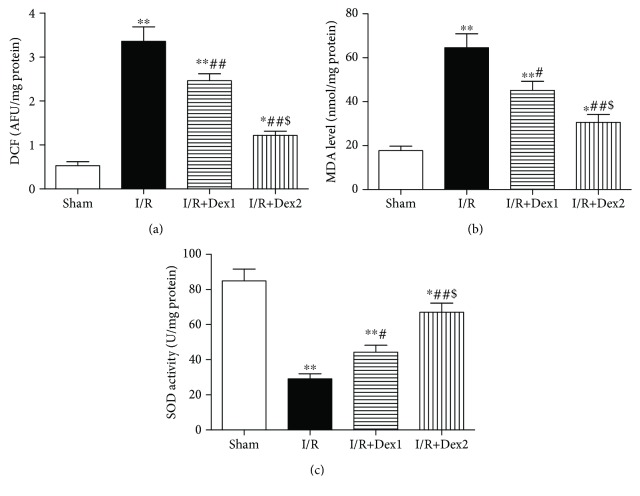
Dexmedetomidine attenuated hepatic oxidative stress. Dexmedetomidine treatment decreased the contents of (a) ROS and (b) MDA and increased (c) SOD activity. The results were presented as the mean ± standard deviation (*n* = 8; ^∗^*P* < 0.05, ^∗∗^*P* < 0.01 vs. sham group; ^#^*P* < 0.05; ^##^*P* < 0.01 vs. I/R group; ^$^*P* < 0.05 vs. I/R+Dex1 group).

As shown in Figure 4, ROS (a) and MDA (b) contents in liver tissue were elevated in the I/R group compared with the sham group (*P* < 0.01). However, dexmedetomidine reduced those levels (*P* < 0.05). In addition, SOD activity (c) in the sham group differed significantly from the lower levels observed in both the I/R and dexmedetomidine treatment groups (*P* < 0.05).

### 3.5. Dexmedetomidine Improved the Expressions of Bax, Bcl-2, and Cleaved Caspase-3 and Decreased Hepatocyte Apoptosis

**Figure 5:**
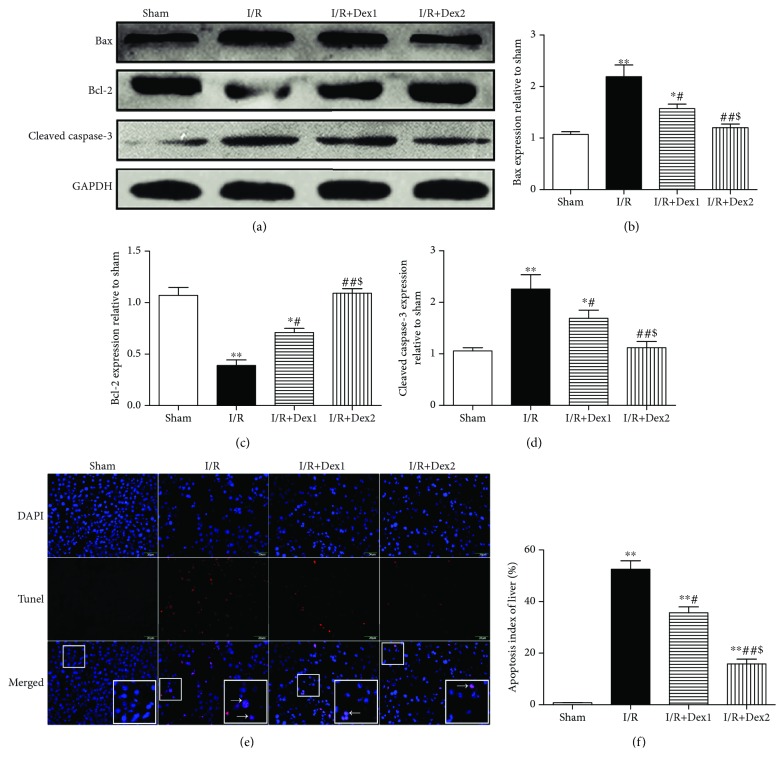
Dexmedetomidine improved hepatic Bax, Bcl-2, and cleaved caspase-3 expressions and reduced liver apoptosis. Dexmedetomidine treatment downregulated cleaved caspase-3 and Bax expressions and upregulated Bcl-2 expression in the liver after intestinal I/R in different groups. Moreover, TUNEL immunofluorescent labelling in the liver. Dexmedetomidine reduced the apoptosis index of the liver (magnification ×400, scale bars = 20 *μ*m; inset, magnified photographs). TUNEL (red) and nuclei (blue) staining were performed after 2 h of reperfusion. The pink in the merged images of red and blue fluorescence indicates TUNEL-positive cells (white arrow).The results were presented as the mean ± standard deviation (*n* = 8; ^∗^*P* < 0.05, ^∗∗^*P* < 0.01 vs. sham group; ^#^*P* < 0.05; ^##^*P* < 0.01 vs. I/R group; ^$^*P* < 0.05 vs. I/R+Dex1 group).

As shown in Figure 5, intestinal I/R induced higher expressions of Bax and cleaved caspase-3 and lower expression of Bcl-2 (*P* < 0.01). Dexmedetomidine downregulated the expression of Bax and cleaved caspase-3, whereas it further upregulated the expression of Bcl-2, especially at the high dose (*P* < 0.05) (a-d). In addition, no TUNEL-positive cells were found in the sham group; yet, positive cells comprised 52.50% of the total cell population in the I/R group. The apoptosis index was lower in the dexmedetomidine treatment groups than in the I/R group (*P* < 0.05) (e-f).

### 3.6. Dexmedetomidine Increased the Levels of Serum and Hepatic Irisin

**Figure 6:**
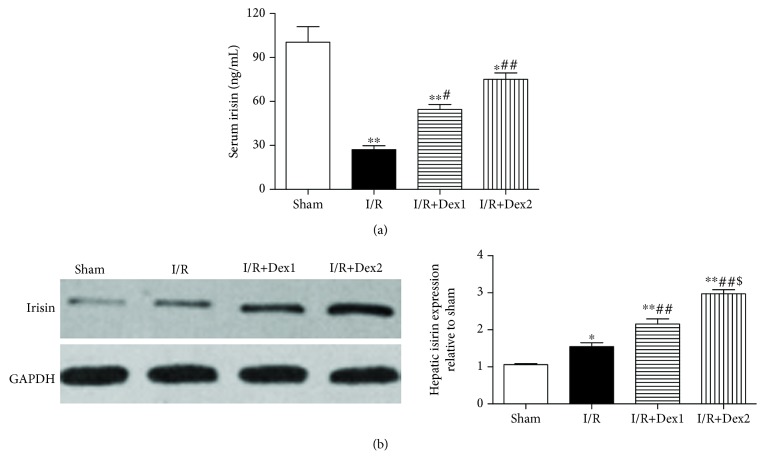
Dexmedetomidine improved the serum and hepatic irisin levels. Dexmedetomidine treatment increased the levels of irisin in the (a) serum and (b) liver. The results were presented as the mean ± standard deviation (*n* = 8; ^∗^*P* < 0.05, ^∗∗^*P* < 0.01 vs. sham group; ^#^*P* < 0.05; ^##^*P* < 0.01 vs. I/R group; ^$^*P* < 0.05 vs. I/R+Dex1 group).

Reduced serum (a) and increased hepatic irisin (b) levels were observed in rats with intestinal I/R compared with the sham group (*P* < 0.05). Furthermore, dexmedetomidine improved these levels in the dexmedetomidine treatment groups compared with the I/R group (*P* < 0.05); most significant changes were observed in the I/R+Dex2 group (Figure 6).

### 3.7. Correlation Analysis of the Serum Irisin Levels with the Liver Injury Scores or TNF-*α* and IL-6 Levels

**Figure 7:**
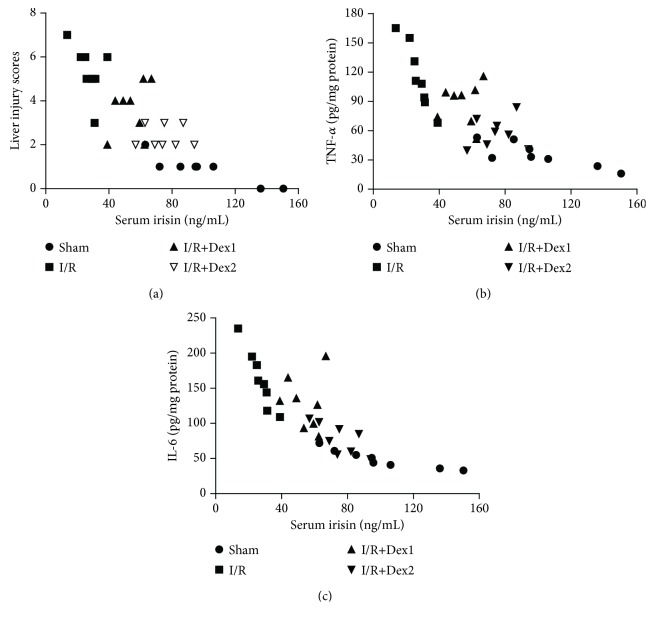
Correlation analysis. Correlations (*N* = 32) between the serum irisin levels and the liver injury scores (a), the serum irisin levels and the hepatic TNF-*α* levels (b), and the serum irisin levels and the hepatic IL-6 levels (c).

Overall (*n* = 32), negative correlations between the serum irisin levels and the liver injury scores (*r* = −0.8862, *P* < 0.05, Figure 7(a)) were found. In addition, negative correlations between the serum irisin levels and the liver TNF-*α* or IL-6 levels were also observed (TNF-*α*, *r* = −0.9363, *P* < 0.01, Figure 7(b); IL-6, *r* = −0.8081, *P* < 0.05, Figure 7(c)).

### 3.8. Effect of Irisin-Neutralizing Antibody on the Levels of Serum and Hepatic Irisin

**Figure 8:**
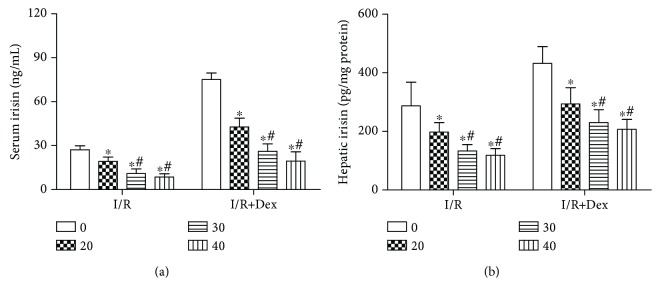
Effect of irisin-neutralizing antibody on the levels of serum and hepatic irisin in I/R and I/R with Dex rats. Irisin-neutralizing antibody treatment reduced the levels of serum (a) and hepatic (b) irisin. The results were presented as the mean ± standard deviation (0: irisin-neutralizing antibody 0 *μ*g; 20: irisin-neutralizing antibody 20 *μ*g; 30: irisin-neutralizing antibody 30 *μ*g; 40: irisin-neutralizing antibody 40 *μ*g) (*n* = 8; ^∗^*P* < 0.05 vs. 0 *μ*g; ^#^*P* < 0.05 vs. 20 *μ*g).

All doses of irisin-neutralizing antibody (NA) reduced serum (a) and hepatic irisin (b) levels in I/R and I/R with Dex rats. Furthermore, most significant changes were observed in the 30 *μ*g and 40 *μ*g. The results showed that irisin NA significantly could reduce serum and hepatic irisin levels to a certain extent in a dose-dependent manner. The dose of 30 *μ*g was relatively appropriate (Figure 8).

### 3.9. Irisin Is Critical for the Hepatoprotection of Dexmedetomidine against Intestinal I/R

**Figure 9:**
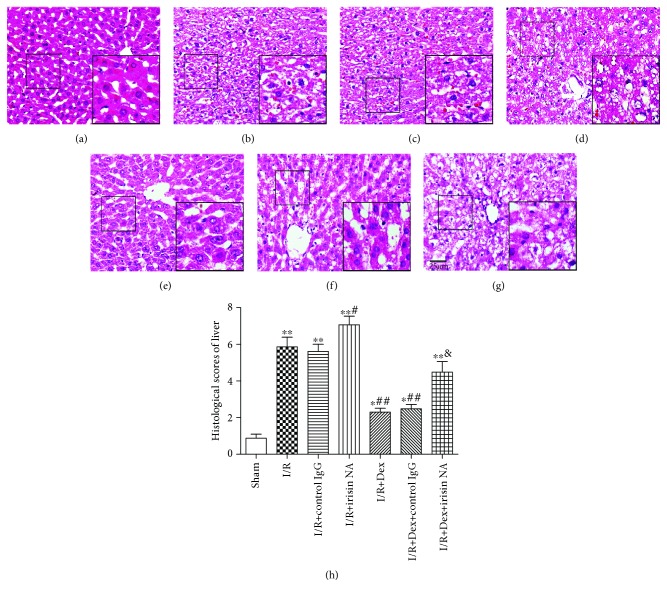
Irisin contributed to the hepatoprotection of dexmedetomidine against intestinal I/R. (a-h) Effect of administration of irisin-neutralizing antibody (irisin NA) on the liver injury (hematoxylin and eosin staining, magnification ×400, scale bars = 25 *μ*m; inset, magnified photographs) (*n* = 8; ^∗^*P* < 0.05, ^∗∗^*P* < 0.01 vs. sham group; ^#^*P* < 0.05; ^##^*P* < 0.01 vs. I/R group; ^&^*P* < 0.05 vs. I/R+Dex group).

**Figure 10:**
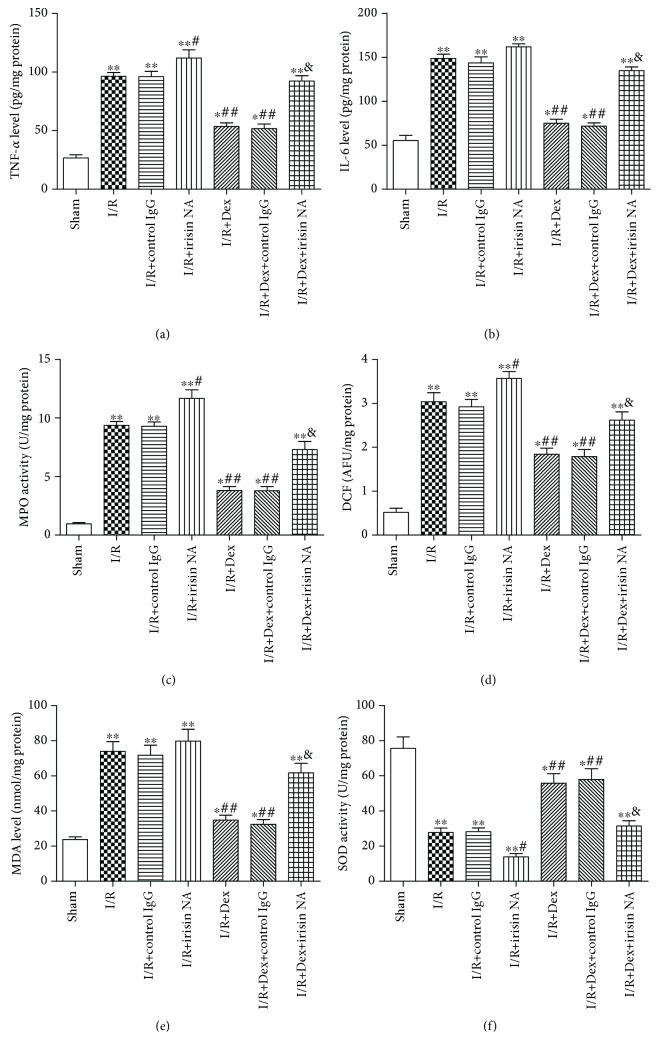
Irisin mediated the protection of dexmedetomidine against hepatic inflammation and oxidative stress injury. Effect of administration of irisin NA on (a) TNF-*α*, (b) IL-6, (c) MPO, (d) ROS, and (e) MDA levels and (f) SOD activity. The results were presented as the mean ± standard deviation (*n* = 8; ^∗^*P* < 0.05, ^∗∗^*P* < 0.01 vs. sham group; ^#^*P* < 0.05; ^##^*P* < 0.01 vs. I/R group; ^&^*P* < 0.05 vs. I/R+Dex group).

Furthermore, we explored the role of irisin in the hepatoprotection of dexmedetomidine against intestinal I/R-induced inflammation and oxidative stress. Dexmedetomidine significantly reduced liver injury (*P* < 0.05). However, this therapeutic effect of dexmedetomidine was substantially alleviated by irisin-neutralizing antibody (irisin NA) (*P* < 0.05), but not by control IgG. The scores of hepatic injury in the I/R+irisin NA group were even significantly higher than that in the I/R+control IgG group (*P* < 0.05) (Figures 9(a)–9(h)). Similarly, dexmedetomidine reduced the TNF-*α*, IL-6, MPO, ROS, and MDA levels and increased SOD activity in the liver tissue of rat with intestinal I/R; however, these effects were partially blocked by irisin NA (*P* < 0.05). Irisin NA treatment resulted in a significant increase in inflammation or oxidative stress injury (*P* < 0.05) (Figures 10(a)–10(f)).

### 3.10. Dexmedetomidine Inhibited NLRP3 Inflammasome Activation via Irisin

**Figure 11:**
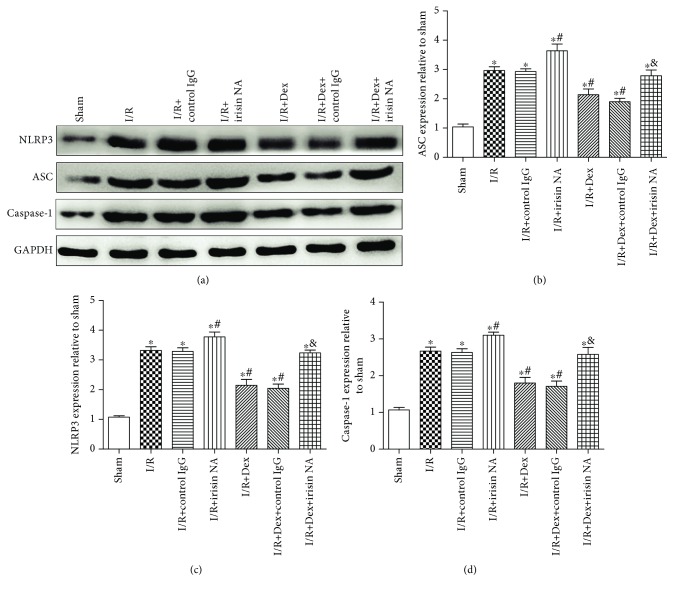
Dexmedetomidine inhibited NLRP3 inflammasome activation via irisin. Effect of administration of irisin NA on NLRP3, ASC, and caspase-1. The results were presented as the mean ± standard deviation (*n* = 8; ^∗^*P* < 0.05, ^∗∗^*P* < 0.01 vs. sham group; ^#^*P* < 0.05; ^##^*P* < 0.01 vs. I/R group; ^&^*P* < 0.05 vs. I/R+Dex group).

As presented in Figure 11, Western blots showed that weak positive signals of NLRP3 inflammasome (NLRP3, ASC, and caspase-1) protein expressions were found in the liver tissues of the sham group. Significant increases of the intensity of NLRP3, ASC, and caspase-1 protein expressions were seen in the I/R group. After dexmedetomidine treatment, the expressions of NLRP3, ASC, and caspase-1 were dramatically reduced following the I/R event (*P* < 0.05). Interestingly, the effect of dexmedetomidine was also attenuated in part by irisin NA (*P* < 0.05), but not by control IgG (a-d).

## 4. Discussion

In the present study, we presented several major findings. First, we found that plasma irisin levels and hepatic Bcl-2 expression decreased, whereas hepatic irisin, cleaved caspase-3, Bax, NLRP3, ASC, and caspase-1 expressions increased following intestinal ischemia reperfusion. Secondly, the results of correlation analyses demonstrated that lower plasma irisin levels were associated with more serious liver damage and higher levels of hepatic proinflammatory cytokines in rats with intestinal I/R. Finally, dexmedetomidine could significantly alleviate intestinal I/R-mediated liver injury, at least in part, by inhibiting NLRP3 inflammasome activation and via regulating irisin expression. The results suggested that irisin is a critical hormone against liver injury in intestinal I/R, which underlies the anti-inflammatory and antioxidative effects of dexmedetomidine.

Intestinal ischemia can easily lead to a series of complications, including intestinal flora shift and cell necrosis, resulting in serious inflammatory reactions and other severe diseases. Intestinal I/R is a severe clinical event associated with high morbidity and mortality. It often follows mesentery embolism, intestinal sepsis, hemorrhagic shock, and transplantation and is thought to be the main inducer of multiple organ dysfunction syndrome [31, 32]. Intestinal I/R leads to local intestinal tissue injury and mucosal barrier dysfunction [33–35] and also causes distant organ dysfunction, especially in the liver and lung [7–9]. Certain studies have suggested that the liver is the first organ to be damaged following the intestinal I/R, because its vasculature is coupled with the intestinal circulation [9, 11]. In addition, blood content in the liver is significantly higher than in other organs, accounting for about 14% of the total human blood volume. Hence, the liver is extremely sensitive to I/R injury, and the damage is more serious compared with other organs [9]. This study demonstrated that the liver was severely damaged after intestinal I/R, including functional and structural damage.

The levels of many adipokines and myokines in the tissues and blood can be affected by cerebral, intestinal, or myocardial ischemia reperfusion [14, 36]. For example, plasma adiponectin levels have shown to be downregulated in subjects with cerebral infarction, whereas plasma visfatin levels are elevated in ischemic stroke. Interestingly, adiponectin is also reduced in the serum of rats with intestinal I/R injury [34]. Therefore, these suggest that irisin levels might be affected by a large variety of factors. Irisin can improve many diseases with evident oxidative stress, inflammation, and apoptosis. In this study, we also found that serum irisin levels are negatively correlated with inflammation. Since irisin is involved in body weight maintenance and energy expenditure, the present study focused on how irisin levels changed in plasma and liver tissues in rats treated with dexmedetomidine.

Many studies have also showed that the plasma irisin concentration in normal human or animals is higher, while the irisin level in pathological groups decreases. Moreover, some researchers suggest that the plasma irisin level might be a marker of organ damage [37–40]. In addition, a recent study has revealed that the plasma irisin concentration decreased in mice with ischemic stroke. The animals with elevated blood irisin levels have a relatively lower degree of cerebral damage, suggesting that irisin could exert neuroprotective effects [16]. Intriguingly, serum irisin levels can be elevated by not only exercise but also cold exposure, limb remote ischemic preconditioning (RIPC), and some drugs [29, 41, 42]. Therefore, it is widely believed that irisin really exists and plays crucial roles in the pathophysiology of many diseases. Here, our results showed that irisin was present in the serum and liver, and this is similar to the results reported by previous studies [13, 43]. Previous study has shown that acute exercises can increase blood irisin levels; however, circulating irisin levels decrease with diabetes and obesity [44]. Recent reports have shown that serum irisin levels decrease after myocardial infarction in rats or pulmonary ischemia/reperfusion injury in mice [15, 41]. It has been demonstrated that the irisin level and NLRP3 inflammasome activation are positively correlated with inflammation and that the production of them can be stimulated by inflammatory substances. In this study, we found that plasma irisin decreased after intestinal I/R, which suggests that the irisin released into the blood stream could be largely suppressed by intestinal I/R. The levels of liver tissue irisin were markedly increased among the three groups compared with those in the sham group. The reason why irisin levels decreased in plasma and increased in liver tissues with intestinal I/R remains unclear. However, the present and previous studies provided the following possible explanations: (a) the animals with brain, intestine, or heart ischemia injury have decreased movements and therefore reduced serum irisin levels [16]; (b) the liver could take up irisin from the extracellular environment under I/R conditions. A recent study has showed that limb remote ischemic preconditioning (RIPC) may cause an increase of irisin in the bloodstream and transfer to injured lung [41]. In the present study, we have explored the effect of dexmedetomidine on the levels of serum and hepatic irisin after I/R injury in rats. The results showed that plasma irisin decreased and hepatic irisin increased after intestinal I/R event; however, dexmedetomidine pretreatment could cause an increase of irisin. It is suggested that plasma irisin might be consumed or the possibility of irisin transferred from the serum to the injured liver after I/R injury. This phenomenon is in agreement with a previous study [45]. The study showed that silencing sulfiredoxin1, an endogenous antioxidant protein, resulted in a significant increase in cerebral histological injury, neurological deficits, and oxidative stress injury after ischemic stroke.

Dexmedetomidine is an *α*_2_-adrenergic agonist with sedative and analgesic effects, which does not cause respiratory depression. It is widely used in clinical practice, especially in the operating rooms and intensive care units [22, 46]. In surgery, dexmedetomidine is always used as an adjuvant drug. It has a sedative effect and it reduces the dose of anesthetic drugs and maintains hemodynamic stability. Recent studies have focused more on the effects of dexmedetomidine on organ protection. Dexmedetomidine can provide neuroprotective and myocardial protection [23, 47]. Furthermore, dexmedetomidine can effectively reduce the intestinal injury induced by intestinal ischemia-reperfusion in rats [24, 28]. The present study showed that dexmedetomidine could reduce the intestinal and hepatic damage caused by intestinal I/R. Dexmedetomidine increased the irisin levels in blood circulation, whereas it or I/R made irisin to transfer from the bloodstream to the injured liver tissue. The transfer of irisin from blood circulation to the liver tissue might underlie the protective function of dexmedetomidine. Dexmedetomidine could attenuate the levels of ALT, AST, and LDH in serum after intestinal I/R and improve liver function. At the same time, it reduced the pathological scores of the liver and intestine tissues. The data suggested that continuous use of dexmedetomidine 60 min before surgery could significantly reduce intestinal I/R-induced intestinal and hepatic injury.

Previous studies have suggested that different doses of dexmedetomidine have different effects on intestinal I/R injury [25, 28]. A low dose (2.5 *μ*g/(kg·h)) of dexmedetomidine did not confer intestinal protection, while a higher dose (10 *μ*g/(kg·h)) led to severe hemodynamic instability. The medium dose (5 *μ*g/(kg·h)) was the optimal dose for intestinal protection and was previously used to decrease inflammatory responses and mortality in septic rats. In the present study, two doses of dexmedetomidine 2.5 and 5 *μ*g/(kg·h) were selected. The results showed that they both had hepatic and intestinal protection, but 5 *μ*g/(kg·h) dexmedetomidine provided more obvious protection. This indicated that the protective effect of dexmedetomidine on intestinal I/R-induced liver lesions was dose dependent.

A large number of studies have demonstrated that excessive generation of ROS, cytokine/chemokine, activated complement, and neutrophil activation is involved in injury to remote organs following intestinal I/R [8–10, 48]. TNF-*α* and IL-6 are both proinflammatory mediators involved in various stages of the inflammatory response. They not only cause direct tissue damage but also induce the activation of neutrophils. The inflammatory response results in liver tissue hyperemia, hepatic edema, and significant hepatocyte apoptosis. Furthermore, MPO activity represents the degree of neutrophil infiltration, and in the present study, it was decreased by dexmedetomidine. Meanwhile, the liver is particularly vulnerable to overproduced ROS. The results from previous studies have indicated that excessive free radicals might lead to lipid peroxidation and induce damage to the membranes of the cell and mitochondria, eventually causing cell apoptosis and necrosis. MDA can reflect the extent of lipid peroxidation in tissues. Moreover, SOD activity may reflect its functional status of scavenging oxygen free radicals. The present study showed that dexmedetomidine could decrease the levels of TNF-*α* and IL-6, reduce ROS and MDA levels, and restore SOD activity in the liver. Additionally, the hepatocyte apoptosis was obvious in the intestinal I/R-induced liver injury, as evidenced by increases in the apoptotic index and cleaved caspase-3 protein expression, while dexmedetomidine could decrease the apoptosis. Some reports have indicated that the antiapoptotic effect of dexmedetomidine might be associated with a reduction in oxidative stress or inflammation, which inhibits the activation of extrinsic apoptotic cascade [24, 26, 49–51].

The important finding in the present study is the important role of irisin in the hepatoprotection of dexmedetomidine. Our results showed that the reduction in the liver injury by dexmedetomidine in the intestinal I/R model is remarkably attenuated by pretreatment with a neutralizing antibody (NA) that targets irisin. Moreover, the therapeutic effects of dexmedetomidine on inflammation, neutrophil infiltration, and oxidative stress were partially blocked by treatment with the irisin NA. Thus, these results revealed that irisin played a crucial role in the beneficial effects of dexmedetomidine on the liver injury after intestinal I/R. We conjectured that irisin might be one of the important endogenous mediators mediating the protection of dexmedetomidine.

There were several limitations in the present study. Firstly, we did not confirm the exact role of dexmedetomidine (direct or indirect effects) in the protection against liver lesions after intestinal I/R. It has been showed that dexmedetomidine can protect against intestinal injury in septic rats by improving intestinal microcirculatory dysfunction [24]. That is, it is possible that the benefits of dexmedetomidine in the liver can partly be attributed to its improvement of microcirculatory function and intestinal injury. Although many studies have demonstrated the protective effects of some interventions on liver injury induced by intestinal I/R, whether they are direct or indirect effects remain unclear [9, 11]. Secondly, the present study did not demonstrate whether hepatic injury and the late elevated levels of inflammatory factors and oxidative stress during intestinal I/R were alleviated by dexmedetomidine. Finally, the present study is an animal research, and the observed findings should be further detected by future studies in humans.

## 5. Conclusion

In the present study, plasma irisin levels decreased and hepatic irisin protein expressions increased after intestinal I/R. Interestingly, lower plasma irisin levels were associated with more serious liver damage and higher levels of hepatic proinflammatory cytokines in rats with intestinal I/R. Dexmedetomidine could significantly alleviate intestinal I/R-mediated liver injury, inflammation, and oxidative stress by inhibiting NLRP3 inflammasome activation, at least in part, via modulating irisin expression.

## Data Availability

The data used to support the findings of this study are included within the article.
